# Development of a microarray-based method for allergen-specific IgE and IgG4 detection

**DOI:** 10.1186/s12014-016-9136-7

**Published:** 2017-01-09

**Authors:** Guzel Feyzkhanova, Sergei Voloshin, Olga Smoldovskaya, Alla Arefieva, Marina Filippova, Viktor Barsky, Ludmila Pavlushkina, Veronika Butvilovskaya, Alexei Tikhonov, Yuri Reznikov, Alla Rubina

**Affiliations:** Engelhardt Institute of Molecular Biology, Russian Academy of Sciences (EIMB RAS), Vavilova str., 32, Moscow, Russia 119991

**Keywords:** Immunoassay, Allergy diagnostics, Microarrays, sIgG4, Biochips

## Abstract

**Background:**

sIgE and sIgG4 detection is necessary for more accurate and effective type I hypersensitivity diagnostics and the estimation of disease development. Typically, the analyses of these antibodies are performed separately with the help of various specialized systems. The aim of this study was to develop a microarray-based method for the simultaneous quantitative detection of sIgE and sIgG4 to the most common allergens in a single sample.

**Methods:**

A quantitative method for the simultaneous detection of sIgE and sIgG4 was developed based on the technology of hydrogel microchips previously designed at Engelhardt Institute of Molecular Biology, Russian Academy of Sciences (EIMB RAS). The microarray contained gel pads with immobilized allergens and gel pads that allow for the obtaining of sIgE and sIgG4 internal calibration curves for each allergen during the assay. The possibility of the simultaneous detection of sIgE and sIgG4 was developed using the corresponding Cy5 and Cy3 fluorescent dyes.

**Results:**

The multiplex immunoassay method using hydrogel microarrays developed in this study allowed the quantitative detection of sIgE and sIgG4 to 31 allergens from different groups in a single assay. A comparison of the microarray with the existing plate-based analogues (i.e., ALLERG-O-LIQ and sIgG4 ELISA) was performed by analysing 152 blood serum samples and by evaluating Pearson correlation coefficients, ROC analysis, and Passing-Bablok linear regression results.

**Conclusion:**

The implementation of this method in allergy diagnostics will provide the possibility of simultaneously performing primary patient screening and obtaining additional information concerning the severity of the allergies and the choice of an appropriate therapy.

## Background

The incidence of allergic diseases steadily increases year after year. One or more allergic signs are detected in 30–40% of the world population, and the number of patients suffering from immunoglobulin-related food intolerance (so-called food allergies) has reached 240–550 million people [1].

According to the classification of Coombs and Gell [2], IgE is assumed to be a key marker of type I hypersensitivity for in vitro diagnostics. Fc region of IgE coupled with allergen has the ability to bind to FcεRI receptor on the basophils and mast cells membranes, that elicits their subsequent activation. This process explains the rapid effects of allergen-sIgE complexes in the formations of allergic rhinitis, asthma, urticaria and anaphylactic reactions [3].

However, the presence of sIgE in the blood is not an absolute marker for the presence of clinical manifestations of immunological failure [4]. In certain cases, the manifestation of allergy symptoms can be suppressed by the presence of immunoglobulin G, which acts as a “blocking antibody”. Typically, such blocking properties are distinctive hallmarks of the IgG4 antibody subclass [5]. According to the number of studies sIgG4 level as well as the ratio sIgE/sIgG4 is associated with likelihood of allergic symptoms reporting [6, 7] and can improve the prediction of tolerance to some allergens [8]. Therefore, sIgG4 detection together with sIgE measurement gives more inclusive information for in vitro analysis interpretation.

The sIgG4 level is also used to monitor allergen-specific immunotherapy (SIT) because successful therapy is characterized by an increase in sIgG4 and a decrease in sIgE [5, 9]. Thus, patient management strategy, particularly the selection of appropriate drugs for SIT, depends not only on the early recognition of the allergens that cause hypersensitivity but also on the monitoring of sIgE and sIgG4 levels [10]. Currently, determination of sIgE in clinical lab is generally performed by numerous singleplex and multiplex tests [11]. Amongst the most useful tools for multiplex sIgE determination are microarrays [12], which allow for the identification of a plurality of analytes during a single analysis of a blood serum sample.

Test systems for the determination of sIgG4 levels in the serum are less widespread. Given that the IgG4 serum concentration on average is greater than the IgE concentration, these tests often require an additional sample preparation stage, i.e., the pre-dilution of the serum prior to analysis. Consequently, the handling of two separate tests is required for the parallel detection of sIgE and sIgG4 in the same serum sample. This requirement complicates the diagnoses of patients with suspected allergies. To solve this problem, an microarray approach based on developing antibodies that are specific to certain classes of immunoglobulins and labelled with various dyes was proposed [13]. The main advantage of this approach is the ability to multiply the number of defined parameters within a single analysis. In comparison with conventional ELISA microarray format allows to detect sIgE and sIgG4 to the number of allergens, including those sensibilization to which was not suspected and was not exhibited because of the blocking antibodies. Furthermore such testing with microarrays requires appreciably less amount of biomaterial.

The study conducted by Rubina et al. in cooperation with Fooke-Achterrath [14] demonstrated the possibility of simultaneous sIgE and sIgG4 detection using hydrogel biochips. As a result of this approach, in this paper we have developed a method for the simultaneous quantitative analysis of allergen-specific immunoglobulins E and G4 to 31 allergens belonging to different groups. The current study presents the analytical characteristics of the developed method and comparison to the established reference methods after assay of serum samples from patients and healthy donors.

## Methods

### Samples

In our research, we included surplus blood serum samples that remained after routine diagnostic procedures. The samples were obtained from healthy donors (control group) and patients who were referred for sIgE determination by allergologists/pulmonologists for the diagnosis and monitoring of the following disease states: atopic dermatitis, asthma, urticaria, and rhinitis. The object of our research was an age-diverse group (5–65 years old) from the Moscow population.

In total, 152 serum samples were analysed in this study. Among these, 82 samples were from adult patients with suspected allergic diseases, and 15 samples were from healthy donors; these samples were provided by the Federal State Budgetary Institution Polyclinic No. 1 of the Business Administration for the President of the Russian Federation. Additionally, 45 sera samples from children with suspected allergic diseases and 10 sera samples from healthy children who did not have allergies were provided by the Filatov Moscow City Pediatric Clinic No. 13.

The conditions of blood sampling, the delivery, and storage of the samples were identical. The blood for serum isolation was collected via a puncture of the median cubital vein, the serum was separated from the blood corpuscles by centrifugation (3000 rpm, 10 min) within the first 2 h after blood sampling. All of the samples were exposed to a single refrigeration at −45 °C. Samples were delivered in insulated containers with ice packs.

### Design and manufacture of the microarrays

Table 1 provides a list of the allergens (GREER, USA) that were immobilized in the microarray gel pads. The microarray contained elements with allergens belonging to the main groups, i.e., pollen, household, epidermal, food, fungi, and insect venoms.

**Table 1:** List of gel pads in the microarray

*Allergens*			
1	Alder	16	*Dermatophagoides pteronyssinus*
2	Birch	17	*Dermatophagoides farinae*
3	Hazelnut	18	*Alternaria tenius*
4	Oak	19	Egg white
5	Wormwood	20	Cow milk
6	Mugwort	21	α-lactalbumin, cow milk^a^
7	Dandelion	22	β-lactoglobulin, cow milk^a^
8	Bermuda grass	23	Casein, cow milk^a^
9	Orchard grass	24	Codfish
10	Meadow fescue	25	Wheat flour
11	Perennial rye grass	26	Peanut
12	Timothy grass	27	Hazelnut
13	Cultivated rye	28	Carrot
14	Cat dander	29	Apple
15	Dog dander	30	Peach
		31	Cockroach, German
*Controlling gel pads*			
32	Anti-human IgE and anti-human IgG4 antibodies		
33	Empty gel pads		
34–45	IgE + IgG4 (internal calibration curve)		
M	Marker gel pads		

^a^Purified allergen components

**Fig. 1:**
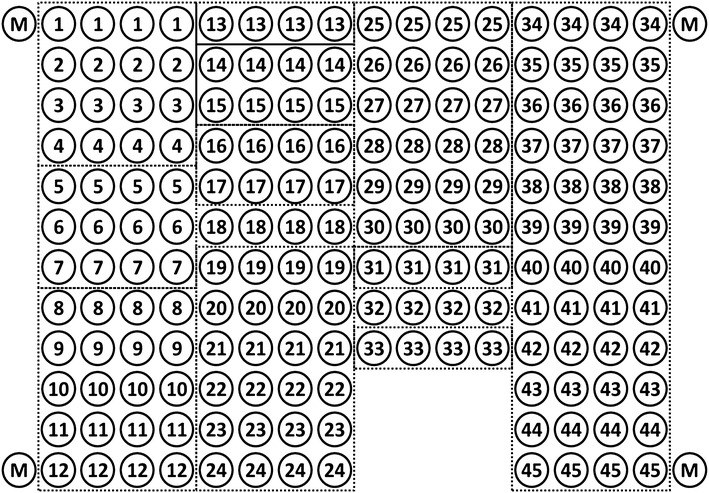
Design of the microarray for the simultaneous detection of sIgE and sIgG4 to 31 allergens. The *numbers* in the *circles* correspond to the allergen numbers in Table 1

**Fig. 2:**
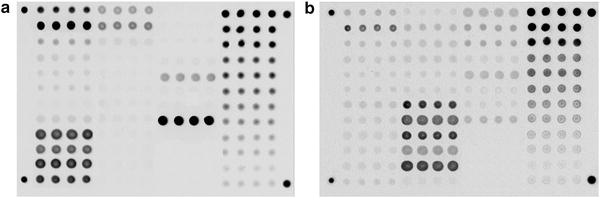
Example microarray fluorescent images after analysis. The images were made after the incubation of the microarray with the serum sample, and development of the fluorescent-labelled anti-human antibodies, anti-IgE-Cy5 and anti-IgG4-Cy3, was achieved via laser excitations at 655 nm (Cy5, **a**) and 532 nm (Cy3, **b**)

Microarray manufacture was performed according to the method developed at Engelhardt Institute of Molecular Biology, Russian Academy of Sciences (EIMB RAS) [15, 16]. For this purpose, glass slides (Menzel-Glaser, Germany) treated with Bind Silane (Amersham Pharmacia Biosciences, USA) were used. The microarray gel pad arrangement is illustrated in Fig. 1. The gel pads contained allergens or mixtures of IgE and IgG4 antibodies at certain concentrations for the internal calibration curve construction (rows 34–45). Immobilized on the glass slide, the proteins were evenly distributed throughout the entire volume (0.1 nl) of each semispherical gel element of approximately 80 microns in diameter. Each probe was immobilized in four repeats to improve the reproducibility of the analysis. Figure 2 provides example microarray images after the performance of the immunoassay.

### Preparation of the dye-conjugated antibodies

Two microliters of Cy5 or Cy3 *N*-hydroxysuccinimide ester solution (GE Healthcare, UK) (2 mg/ml in dimethylformamide) were added to 75 µl of anti-IgE (Bethyl, USA) or anti-IgG4 (Fitzgerald, USA) antibody solution (1 mg/ml in 0.01 M bicarbonate buffer, pH 9.5). The reaction was performed at 22 °C for 1 h in the dark with stirring (550 rpm). Purification of the antibody conjugate from the unreacted dye (Cy5 or Cy3) was performed by gel filtration on a Micro Bio-Spin column (Bio-Rad, USA) filled with Sephadex G-25 Coarse (Sigma, USA) and equilibrated with PBS (0.01 M phosphate buffer, pH 7.2, 0.15 M NaCl) buffer. The dye/protein molar ratio of the final conjugates was determined spectrophotometrically to be 3.

### Analysis of sIgE and sIgG4 on the microarrays

Each microarray was incubated with sixty-five microliters of blood serum at 37 °C for 20 h. After washing in PBST (PBS, 0.1% Tween-20 (Sigma, USA)) for 20 min, 50 µl of developing solution containing anti-IgE-Cy5 and/or anti-IgG4-Cy3 (working concentrations of 2.5 and 1.5 µg/ml, respectively) was applied to the microarray, and the microarray was incubated at 37 °C for 1 h in the dark. After a final washing in PBST for 30 min, the registration of the fluorescence signals was performed.

### Measurement of the fluorescence intensities of the microarray gel pads

The registration of fluorescence signals was performed using a two-wavelength microarray analyser (EIMB RAS) based on the concept of digital wide-field fluorescence microscopy. While working with the fluorescent dyes, excitation was provided by laser diodes that emitted light at 532 nm (Cy3) or 655 nm (Cy5). The fluorescence intensities of the gel elements were registered by a CCD camera using interference filters of 607 ± 35 nm for Cy3 and 716 ± 22 nm for Cy5. The analyser operation, the analyses of the fluorescent images and the calculations of the sIgE and sIgG4 concentrations were performed with *ImageAssay* software (EIMB RAS). A standard method of the gel element fluorescence calculation that has been previously described [17] was employed. The final fluorescence of each data point was calculated as the median value of the four fluorescence signals obtained from the repeats.

### Processing and interpretation of the results

The concentrations of antibodies that were immobilized in the microarray gel pads 34–45 (Fig. 1) were chosen and arranged in ascending order so that after analysing the fluorescent signals from rows 34–45, the entire ranges of the signal intensities for sIgE (up to 100 IU/ml) and sIgG4 (up to 2500 ng/ml) were covered.

Each manufactured lot of microarrays was calibrated using characterized standard blood sera-based samples including a zero sample (PBS, 0.1% PVA, and 0.1% PVP). For each allergen, the sIgE and sIgG4 concentrations of the standard samples were ascribed to the relative fluorescence intensity of each gel pad that was used for calibration curve plotting.

Treatment with a mixture of fluorescently labelled conjugates of anti-IgE-Cy5 and anti-IgG4-Cy3 resulted in the formation of binary complexes with corresponding conjugates in the gel pads 34–45. According to the fluorescent signals from these gel pads and the attributed concentrations of sIgE and sIgG4, internal calibration curves were constructed for each of the allergens. The determinations of the sIgE and sIgG4 concentrations were performed according to the fluorescent signals from the gel pads with the immobilized allergens in relation to the internal calibration curves.

### Analysis of IgE and IgG4 using reference methods

The analyses of the serum samples were performed using the Specific IgE REAST (ALLERG-O-LIQ) and Specific IgG4 ELISA (Dr. Fooke Laboratorien GmbH, Germany) test systems according to the procedures described in the manufacturer’s instructions. Data processing was performed with the ALLERG-O-Win software (Dr. Fooke Laboratorien, GmbH).

### Determination of the analytical characteristics

#### Dilution test

The linearity of the method was evaluated via the analysis of blood serum samples that had been diluted 2, 4, 8, and 16 times. The dilutions were performed with the zero sample.

#### Detection limit

The detection limits for sIgE and sIgG4 were determined via serial dilutions of two samples that contained significant amounts of sIgE to pollen (grey alder, birch, meadow fescue, timothy grass), cat dander, and cow milk and sIgG4 to cat dander, dog dander, cow milk, wheat, peanut, and hazelnut. The detection limit for each allergen was established as the concentration associated with the fluorescence value that was two standard deviations larger than the average value of the tenfold measured fluorescent signal of the zero sample.

#### Within-run precision

The evaluation of the within-run precision of the method was performed via an analysis of blood serum samples containing sIgEs and sIgG4s to various allergens. The assay was performed using 10 repeats for each sample. The samples were chosen such that the concentrations of sIgE and sIgG4 covered the entire dynamic ranges of the measurements.

#### Comparison with other methods: correlation and regression analysis

For a number of allergens, the Pearson correlation coefficients *r* [18] of the concentrations obtained by the microarray and commercial test systems were determined. Passing–Bablok regression analyses [19], ROC curve analysis, sensitivity and specificity were also performed using the MedCalc program, version 16.4.3. The parameters of the regression line were determined as (Y = A + B × X), where the intercept is A, the slope is B, and the associated 95% confidence intervals were calculated.

## Results and discussion

### Design of the microarray and analysis procedure

The three-dimensional hydrogel microarray produced at EIMB RAS was used as an analytical instrument for the development of a multiplex simultaneous quantitative assay of sIgEs and sIgG4s for 31 allergens in blood serum samples.

The microarray structure and lists of the immobilized allergen extracts and purified components are provided in Fig. 1 and Table 1. The allergens were chosen with consideration of the frequencies of allergen reactions in Central Russia, which mainly corresponds to the frequencies in Central and Northern Europe [20]. The allergens belonged to different groups that included pollen, indoor allergens and food allergens.

In addition to allergens, the structure of the microarray was enlarged with gel pads with immobilized immunoglobulins E and G4, which were used to plot an internal calibration curve (after development with Cy5- and Cy3-labeled anti-human antibodies). The internal calibration curve was used to control for the development system activity during the assay.

### Mutual influence of Cy3- and Cy5-labeled antibodies on the developing system

**Fig. 3:**
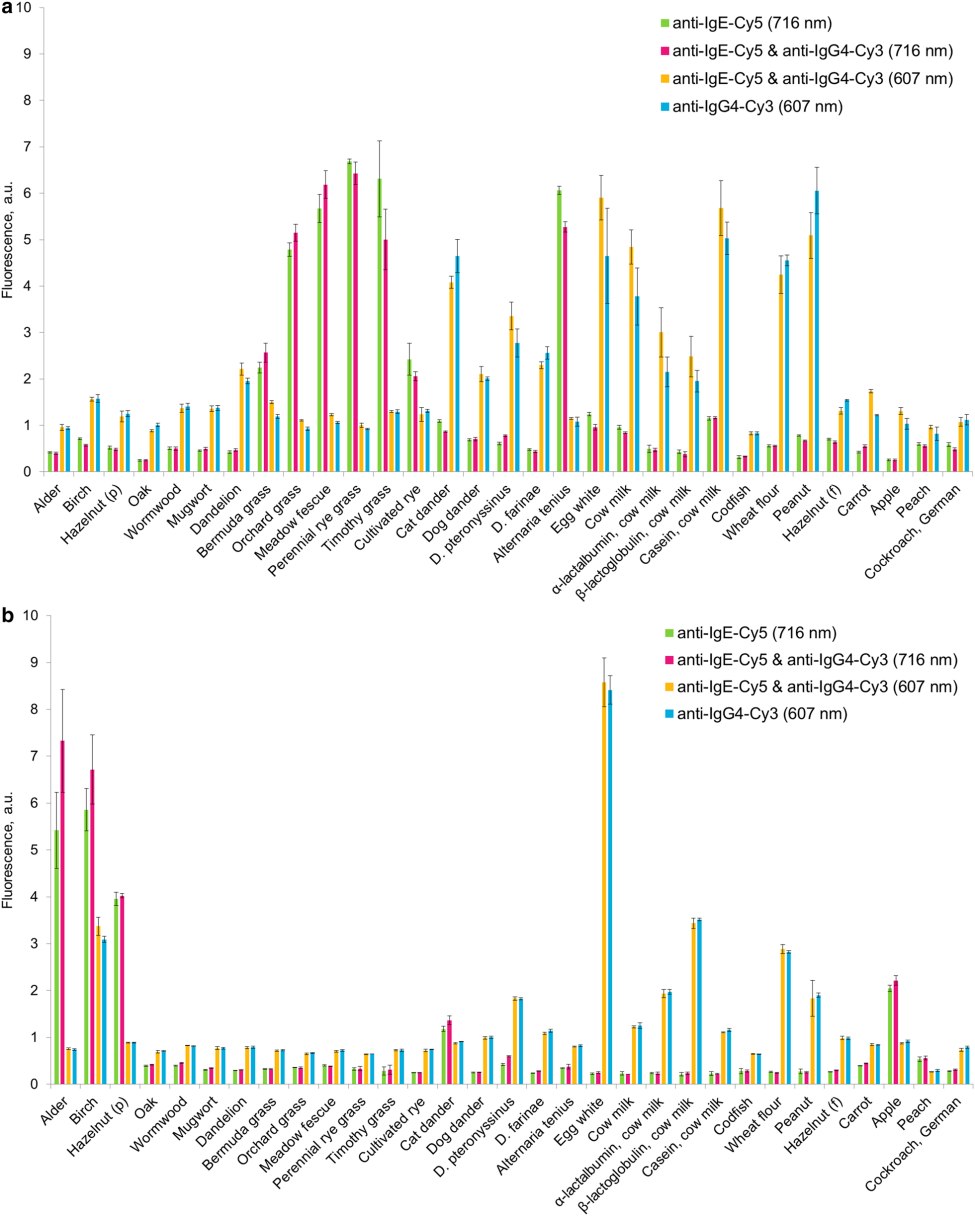
Fluorescent signals obtained using different developing systems for two different samples (**a** and **b**). The fluorescence of Cy5 for sIgE (*green columns*: developed with anti-IgE-Cy5 individually; *red columns*: developed with an anti-IgE-Cy5/anti-IgG4-Cy3 mixture) was registered at 716 ± 22 nm, and the fluorescence of Cy3 for sIgG4 (*yellow columns*: developed with an anti-IgE-Cy5/anti-IgG4-Cy3 mixture; *blue columns*: developed with anti-IgG4-Cy3 individually) was registered at 607 ± 35 nm

To control the validity of the developing system that contained mixtures of antibodies labelled with different fluorescent dyes, an analysis of the serum samples was performed using three variations of developing solutions: anti-IgE-Cy5, anti-IgG4-Cy3, and a mixture of anti-IgE-Cy5 and anti-IgG4-Cy3. The fluorescent signals obtained after the analysis are illustrated in Fig. 3. The differences in the signals of the individually labelled antibodies and their mixture fit within the standard deviation of the analysis of the hydrogel microarrays, which indicates the propriety of the selected developing system.

### Analytical characteristics of the method

The accuracy of the developed method was evaluated via an estimation of analytical characteristics including the dilution linearity, detection limit and within-run precision.

### Dilution linearity

This test was performed via the serial dilution of a sample with a high analyte concentration and follow-up comparisons of the experimental concentrations with the estimated concentrations.

**Fig. 4:**
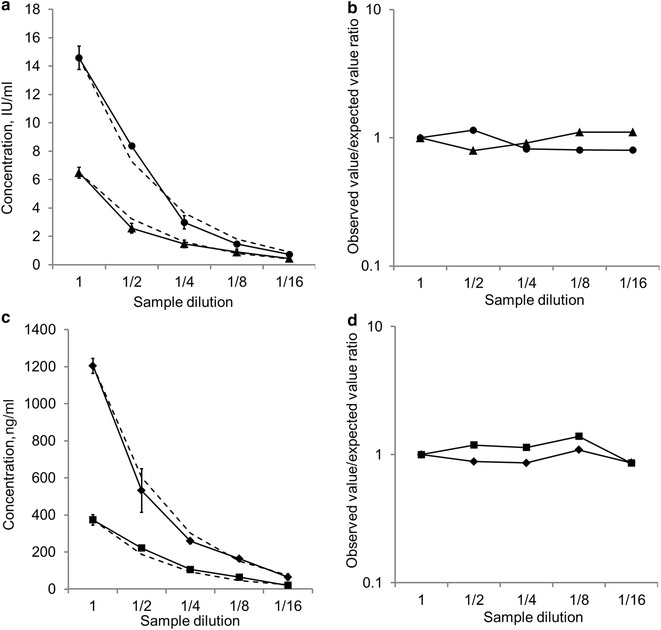
Results of the serum sample dilution analysis. **a** The concentration-dilution curve for the measured (*solid lines*) and expected (*dashed lines*) concentrations of sIgE to birch pollen (*circles*) and meadow fescue pollen (*triangles*). **b** The experimental/expected concentration ratios of sIgE to the allergens listed above. **c** The concentration-dilution curves for the measured (*solid lines*) and expected (*dashed lines*) concentrations of sIgG4 to cow milk (*diamonds*) and peanut (*squares*). **d** The experimental/expected concentration ratios of sIgG4 to the allergens listed above

A blood serum sample containing 14.58 IU/ml sIgE to birch pollen, 6.49 IU/ml sIgE to meadow fescue, 1205 ng/ml sIgG4 to cow milk and 373 ng/ml sIgG4 to peanut was diluted 2, 4, 8, and 16 times with the zero sample. Figure 4a, c illustrate the decreasing concentration-dilution curves for the sIgE and sIgG4 measurements, respectively. The dashed line depicts the corresponding curves for the expected concentrations. The experimental/expected concentration ratios for the different dilutions are provided in Fig. 4b, d for sIgE and sIgG4, respectively.

As illustrated in the figures, the experimentally determined sIgE concentrations differed from the calculated concentration by no more than ±10%. The experimental/expected concentration ratios for sIgG4 were also in the range of 0.9–1.1. The presence of the regular pattern during the serum dilution indicates the absence of a matrix effect, i.e., a lack of interference from different serum components. Consequently, the mean per cent linearities for both sIgE and sIgG4 for the serum were in the range of 90–110%, which meets with the requirements for immunoassay methods.

### Detection limit

The detection limits were determined via serial dilution of the serum samples. For example, the reliable detected concentrations for the serum sample with sIgE concentrations to meadow fescue (15.14 IU/ml), timothy grass (11.11 IU/ml), cat dander (3.36 IU/ml), and cow milk (2.38 IU/ml) were 0.24 IU/ml, 0.17 IU/ml, 0.21 IU/ml, and 0.15 IU/ml, respectively. The same sample contained 708 ng/ml sIgG4 to cat dander and 432 ng/ml sIgG4 to peanut. The reliable sIgG4 concentrations after dilution were 11 and 14 ng/ml, respectively.

For all allergens, the limit of sIgE detection did not exceed 0.25 IU/ml. This value is above the detection limit of the existing methods (0.1 IU/ml for ImmunoCAP and Immulite) but still below the internationally accepted cut-off concentration for allergodiagnostics (0.35 IU/ml). The detection limit for the sIgG4 concentration did not exceed 100 ng/ml.

### Within-run precision

Table 2 provides the average concentrations that were determined in the assay of the 2 serum samples in 10 repetitions and the calculated coefficients of variation for the different allergens. As shown in the table, the within-run precision did not exceed 15% for sIgE or 17% for sIgG4 determinations in the measured concentration range.

**Table 2:** Results of the within-run precision test

sIgE analysis				sIgG4 analysis			
Sample	Allergen	Average sIgE concentration, IU/ml	CV, %	Sample	Allergen	Average sIgG4 concentration, ng/ml	CV, %
#1	Cat dander	0.76	5.7	#1	Hazelnut	112	7.9
#1	Timothy grass	2.77	7.6	#1	Cow milk	529	11.3
#1	Mugwort	32.4	11.5	#1	Wheat	926	15.6
#1	House dust mite (*D. pteronyssinus*)	59.7	14.5	#1	Egg	2165	16.8
#2	Cat dander	0.57	6.7	#1	Hazelnut	108	7.6
#2	Timothy grass	1.56	9.5	#2	Wormwood	27.5	10.1
#2	Mugwort	70.06	13.7	#2	Peanut	74.8	8.4

The average sIgE and sIgG4 concentrations were obtained in a simultaneous immunoassay of the microarrays, and the corresponding coefficients of variation for the 2 serum samples analysed in 10 repetitions are provided

### The results of the comparison between the hydrogel microarrays and ELISA

**Fig. 5:**
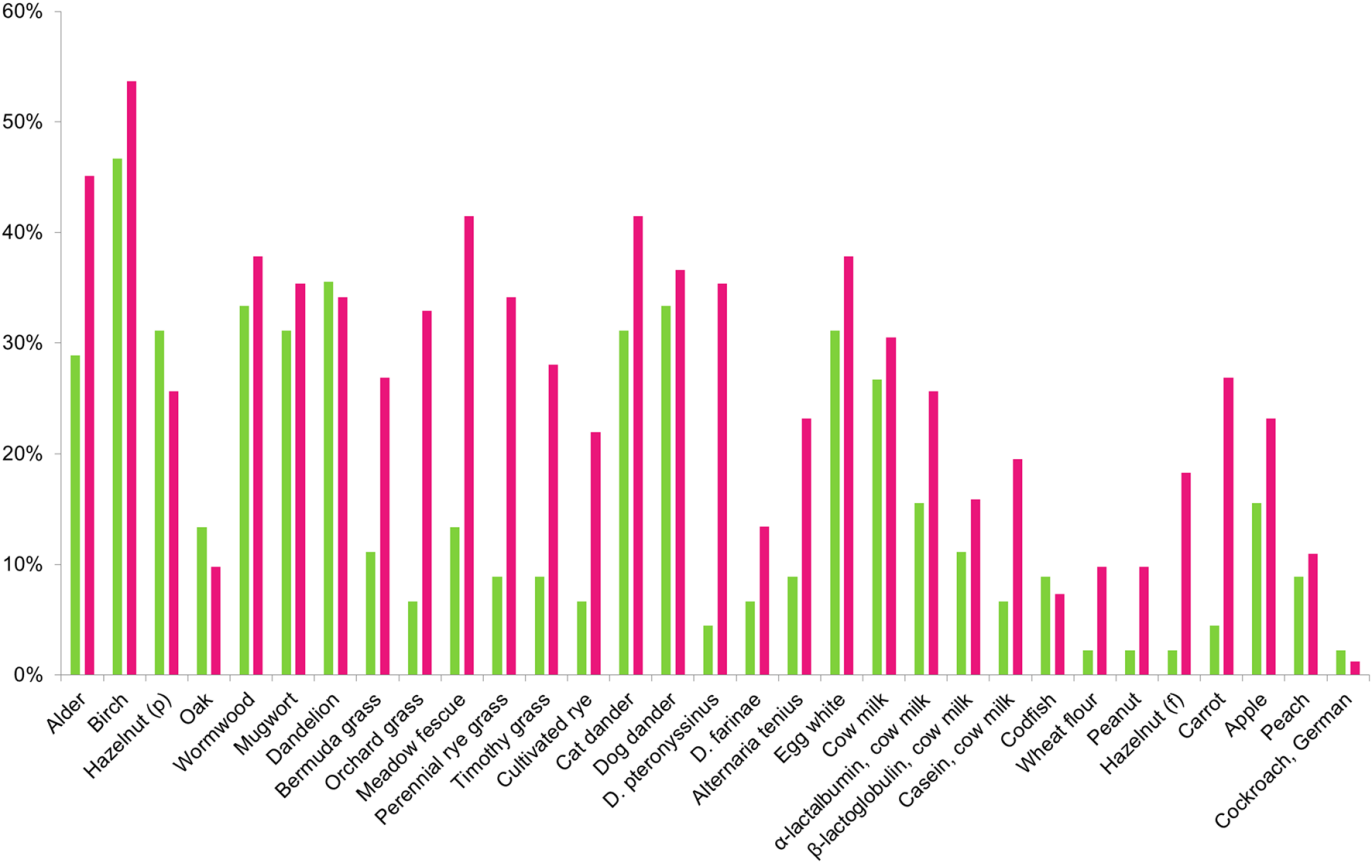
Percentage increases in sIgE concentrations (≥0.35 IU/ml) to the different allergens. The results were obtained after analyses of serum samples from children (*green columns*) and adults (*red columns*) with the hydrogel microarrays

The serum samples (n = 127) were obtained from atopic patients from 2 age groups, i.e., age 0.5–17 years (n = 45) and age 18–74 years (n = 82). Figure 5 illustrates the distribution of increased (≥0.35 IU/ml) sIgE concentrations for different allergens for the patients based on the experimental data. For the comparative evaluation of the multiplex simultaneous sIgE and sIgG4 immunoassay on the hydrogel microarrays, analyses of 152 serum samples (127 atopic patients and 25 healthy donors) for different allergens were performed with both the microarrays and the reference methods for the sIgE (ALLERG-O-LIQ) and sIgG4 (sIgG4 ELISA) determinations. For each allergen, no fewer than 10 serum samples were analysed using ALLERG-O-LIQ and an sIgG4 ELISA.

**Fig. 6:**
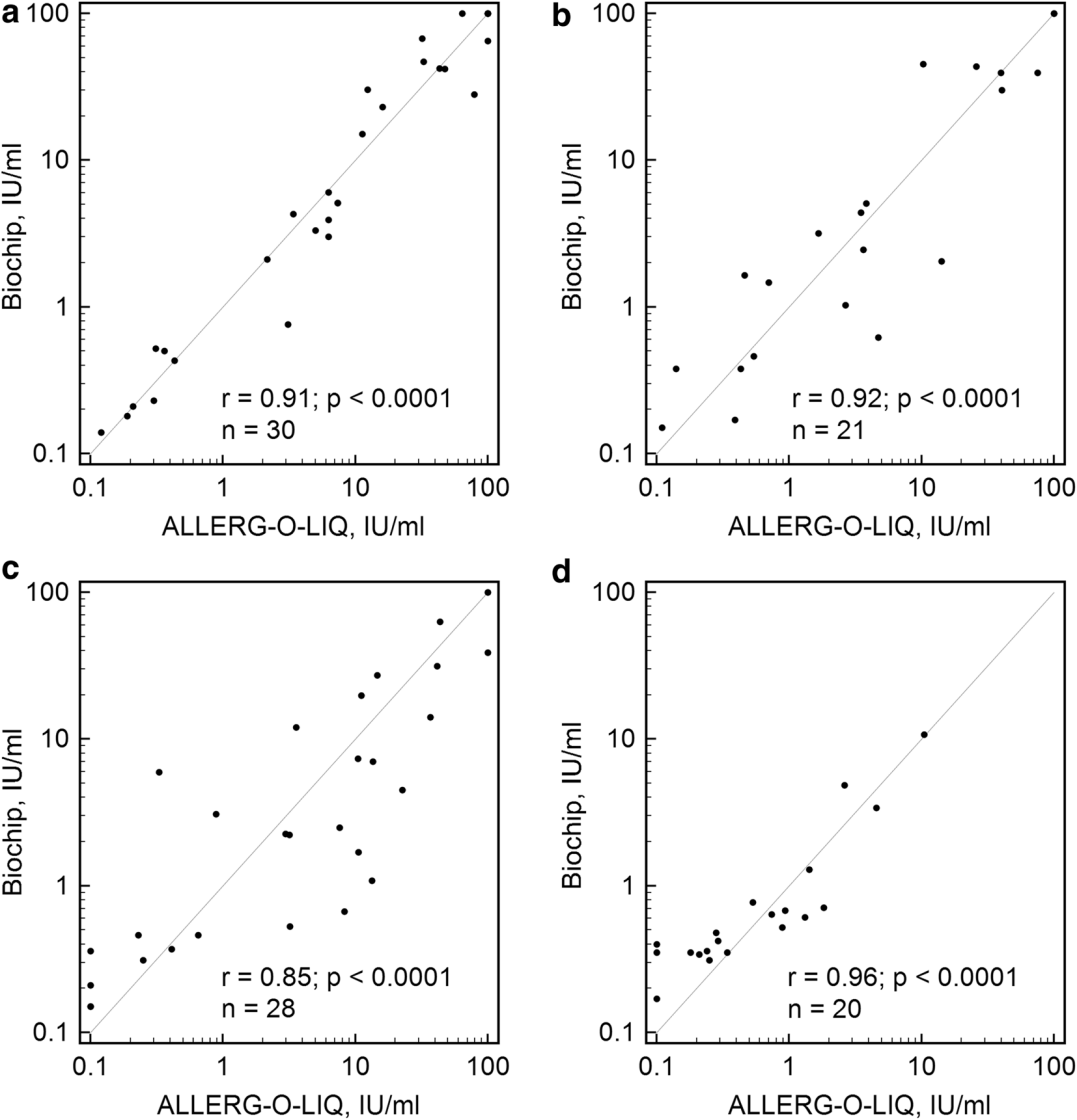
Scatter diagrams of the comparisons of the hydrogel microarray with ALLERG-O-LIQ. Results for the sIgE determinations to the following allergens: **a** birch pollen, **b** mugwort pollen, **c** timothy grass pollen, and **d** dog dander

**Fig. 7:**
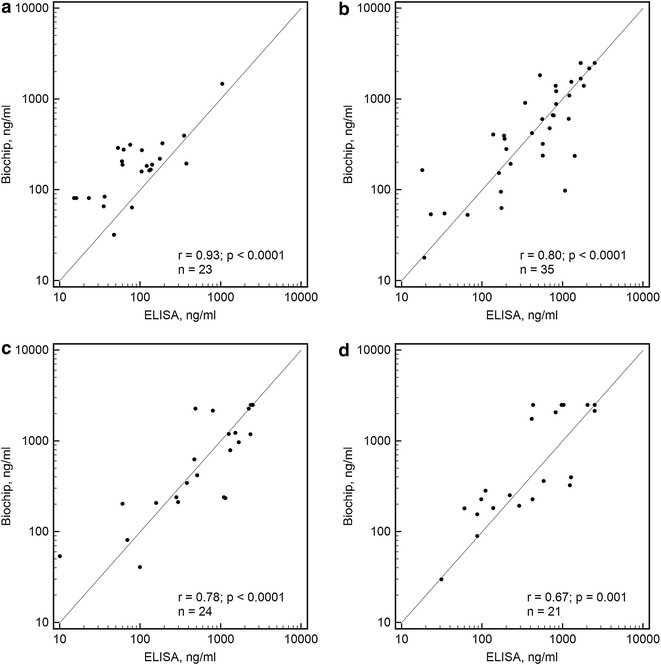
Scatter diagrams for the comparisons of the hydrogel microarray with the sIgG4 ELISA. Results for the sIgG4 determinations to the following allergens: **a** dog dander, **b** cow milk, **c** wheat flour, and **d** hazelnut (food)

Scatter diagrams of the results obtained from the analyses of sIgE concentrations to birch pollen, mugwort pollen, timothy grass pollen, and dog dander on the hydrogel microarrays and ALLERG-O-LIQ are provided in Fig. 6. The scatter diagrams of the results for the sIgG4 concentrations to dog dander, cow milk, wheat flour, and hazelnut in the samples as measured on the hydrogel microarrays and the sIgG4 ELISA are provided in Fig. 7.

Pearson’s correlation coefficients *r* and the sample sizes *n* for the analysed allergens are provided in Table 3. For the remaining allergens, sufficient sample sizes were not obtained for the determination of correlation coefficients with p < 0.01.

**Table 3:** Microarray: ALLERG-O-LIQ and microarray—sIgG4 ELISA comparisons by Passing-Bablok regression

Allergen	sIgE or sIgG4	n^a^	r^b^	*p* value	Intercept A	95% confidence interval	Slope B	95% confidence interval
Dog dander	sIgG4	23	0.93	<0.0001	35.07	−78.62 to 65.86	1.36	0.95–2.35
Egg white	sIgG4	22	0.84	<0.0001	59.13	11.09–134.25	0.82	0.60–1.19
Cow milk	sIgG4	35	0.80	<0.0001	−11.73	−121.93 to 57.59	1.02	0.86–1.30
Wheat flour	sIgG4	24	0.78	<0.0001	−9.89	−119.54 to 55.06	0.99	0.76–1.09
Peanut	sIgG4	27	0.79	<0.0001	50.98	−62.00 to 168.13	1.95	0.93–2.74
Hazelnut	sIgG4	21	0.67	0.001	18.79	−150.65 to 144.49	1.19	0.61–2.58
Alder	sIgE	21	0.67	0.0009	−0.45	−1.75 to 0.26	0.83	0.30–1.19
Birch	sIgE	30	0.91	<0.0001	0.00	−0.32 to 0.15	1.00	0.92–1.04
Mugwort	sIgE	21	0.92	<0.0001	0.04	−0.19 to 0.97	0.99	0.71–1.20
Meadow fescue	sIgE	18	0.92	<0.0001	−0.88	−3.62 to 0.56	1.02	0.48–1.75
Timothy grass	sIgE	28	0.85	<0.0001	0.08	−0.52 to 0.27	0.69	0.39–1.04
Cat dander	sIgE	16	0.78	0.0004	0.18	−4.20 to 0.28	0.71	0.49–2.08
Dog dander	sIgE	20	0.96	<0.0001	0.20	0.09–0.31	0.70	0.36–1.01

^a^Sample size

^b^Pearson correlation coefficient

The correlation coefficients observed in the comparisons between the microarray and reference methods for the different allergens were in the ranges of 0.68–0.93 for the sIgE analyses and 0.67–0.96 for the sIgG4 analyses. The obtained values are similar to the values of the correlation coefficients that have been calculated in comparisons of immunoassay methods in other works (for example, r = 0.525–0.979 in [21] and r = 0.60–0.98 in [22]). For some allergens the significant dispersion of the results led to the small correlation coefficients. This fact can be explained by the distinct component compounds of the protein allergen extracts produced by numerous manufacturers, by the variety of protein modifications that occur during the immobilization process, as well as the wide range of developing anti-human antibodies that can interact with sIgE populations with different efficacies [23].

Passing-Bablok regression analyses yielded the intercept A and slope B for each allergen. The values are presented in Table 3. For all except two cases (sIgG4 to egg white and sIgE to dog dander), the 95% confidence intervals for the intercept included 0, and for all cases, the 95% confidence intervals for the slope included 1.

ROC analysis for sIgE detection was performed with the data differentiated through the common sIgE cut-off of 0.35 IU/ml (with disease: ≥0.35 IU/ml sIgE with the reference method; without disease: <0.35 IU/ml sIgE with the reference method). The optimum cut-off for the described microarray-based method as defined by the Youden’s J statistic-associated criterion was 0.52 IU/ml. For this cut-off the sensitivity was 87.6%, the specificity was 90.6%, and the diagnostic accuracy was 87%. The area under the curve (AUC) was 0.931 that corresponded to high accuracy test [24]. In general, the results observed following the application of different immunoassay methods cannot be inter-convertible because of significant differences that inevitably appear during clinical sample assays [25, 26]; however, in our case, it may be said that for these concretely analysed allergens, there is no overestimation or underestimation of the sIgE and sIgG4 measurements compared to the employed reference methods, i.e., ALLERG-O-LIQ and sIgG4 ELISA.

## Conclusion

A microarray for the multiplex quantification of the concentrations of sIgE and sIgG4 to 31 allergens from different groups in serum samples was developed. The simultaneous detection of sIgE and sIgG4 was made possible via the use of a developing system with two fluorescent dyes. This method allows for the obtaining of sIgE and sIgG4 levels in common units without the construction of an external calibration curve. The analytical characteristics of the method satisfy the requirements that are applicable to immunofluorescent test systems.

The usage of this method in allergy diagnostics provides the possibility of both performing primary patient screening and obtaining the additional information that is necessary for allergy severity evaluation and therapy selection.

## Abbreviations

EIMB RAS: Engelhardt Institute of Molecular Biology, Russian Academy of Sciences
sIgE: specific immunoglobulin E
sIgG4: specific immunoglobulin G4
SIT: allergen-specific immunotherapy
Cy5: cyanine 5
Cy3: cyanine 3
PBS: phosphate-buffered saline
PBST: phosphate-buffered saline with Tween^®^ 20
PVA: polyvinyl alcohol
PVP: polyvinylpyrrolidone

## References

[1] World Allergy Organization White Book on Allergy: Update 2013. World Allergy Organization, 2013.

[2] Coombs RR, Gell PG (1975). Classification of allergic reactions responsible for clinical hypersensitivity and disease. Clin Asp Immunol.

[3] Grimbaldeston MA, Metz M, Yu M, Tsai M, Galli SJ (2006). Effector and potential immunoregulatory roles of mast cells in IgE-associated acquired immune responses. Curr Opin Immunol.

[4] Bousquet J, Anto JM, Bachert C, Bousquet PJ, Colombo P, Crameri R, Daeron M, Fokkens W, Leynaert B, Lahoz C, Maurer M, Passalacqua G, Valenta R, Van Hage M, Van Ree R, Daeron M, Fokkens W, Leynaert B, Lahoz C, Maurer M, Passalacqua G, Valenta R, Van Hage M, Van Ree R (2006). Factors responsible for differences between asymptomatic subjects and patients presenting an IgE sensitization to allergens. A GA2LEN project. Allergy Eur J Allergy Clin Immunol.

[5] Greenberger PA (2002). Immunotherapy update: mechanisms of action. Allergy Asthma Proc.

[6] Burnett M, Wegienka G, Havstad S, Kim H, Johnson CC, Ownby D, Zoratti E (2013). Relationship of dog- and cat-specific IgE and IgG4 levels to allergic symptoms on pet exposure. J Allergy Clin Immunol Pract.

[7] Santos AF, James LK, Bahnson HT, Shamji MH, Couto-Francisco NC, Islam S, Houghton S, Clark AT, Stephens A, Turcanu V, Durham SR, Gould HJ, Lack G (2015). IgG4 inhibits peanut-induced basophil and mast cell activation in peanut-tolerant children sensitized to peanut major allergens. J Allergy Clin Immunol.

[8] Vazquez-Ortiz M, Pascal M, Jiménez-Feijoo R, Lozano J, Giner MT, Alsina L, Martín-Mateos MA, Plaza AM (2014). Ovalbumin-specific IgE/IgG4 ratio might improve the prediction of cooked and uncooked egg tolerance development in egg-allergic children. Clin Exp Allergy.

[9] Bullock RJ, Barnett D, Howden MEH (2005). Immunologic and clinical responses to parenteral immunotherapy in peanut anaphylaxis—a study using IgE and IgG4 immunoblot monitoring. Allergol Immunopathol (Madr).

[10] Stylianou E, Ueland T, Borchsenius F, Michelsen AE, Øvstebø R, Mollnes TE, Skjønsberg OH, Aukrust P (2016). Specific allergen immunotherapy: effect on IgE, IgG4 and chemokines in patients with allergic rhinitis. Scand J Clin Lab Invest.

[11] Matricardi PM, Kleine-Tebbe J, Hoffmann HJ, Valenta R, Hilger C, Hofmaier S, Aalberse RC, Agache I, Asero R, Ballmer-Weber B, Barber D, Beyer K, Biedermann T (2016). EAACI molecular allergology user’s guide. Pediatr Allergy Immunol.

[12] Passalacqua G, Melioli G, Bonifazi F, Bonini S, Maggi E, Senna G, Triggiani M, Nettis E, Rossi RE, Vacca A, Canonica GW (2013). The additional values of microarray allergen assay in the management of polysensitized patients with respiratory allergy. Allergy.

[13] Renault NK, Gaddipati SR, Wulfert F, Falcone FH, Mirotti L, Tighe PJ, Wright V, Alcocer MJC (2011). Multiple protein extract microarray for profiling human food-specific immunoglobulins A, M, G and E. J Immunol Methods.

[14] Rubina AY, Feizkhanova GU, Filippova MA, Talibov VO, Fooke-Achterrath M, Zasedatelev AS (2012). Multiplex assay of allergen-specific and total immunoglobulins of E and G classes in the biochip format. Dokl Biochem Biophys.

[15] Rubina AY, Dementieva EI, Stomakhin AA, Darii EL, Pan’kov SV, Barsky VE, Ivanov SM, Konovalova EV, Mirzabekov AD (2003). Hydrogel-based protein microchips: manufacturing, properties, and applications. Biotechniques.

[16] Feyzkhanova GU, Filippova MA, Talibov VO, Dementieva EI, Maslennikov VV, Reznikov YP, Offermann N, Zasedatelev AS, Rubina AY, Fooke-Achterrath M (2014). Development of hydrogel biochip for in vitro allergy diagnostics. J Immunol Methods.

[17] Rubina AY, Filippova MA, Feizkhanova GU, Shepeliakovskaya AO, Sidina EI, Boziev KhM, Laman AG, Brovko FA, Vertiev YV, Zasedatelev AS, Grishin EV (2010). Simultaneous detection of seven staphylococcal enterotoxins: development of hydrogel biochips for analytical and practical application. Anal Chem.

[18] Armitage P, Berry G, Matthews JNS (2002). Statistical methods in medical research.

[19] Passing H, Bablok W (1983). A new biometrical procedure for testing the equality of measurements from two different analytical methods. Application of linear regression procedures for method comparison studies in Clinical Chemistry, Part I. Clin Chem Lab Med.

[20] Reznikov YP, Topoleva TS, Goryackina LA (2001). Composition of “Russian allergologic panel” for in vitro diagnosis of atopia in children and adolescents. J “Pediatria” Named After GN Speransky.

[21] Lee YW, Sohn JH, Lee J-H, Hong C-S, Park J-W (2009). Allergen-specific IgE measurement with the IMMULITE 2000 system: intermethod comparison of detection performance for allergen-specific IgE antibodies from Korean allergic patients. Clin Chim Acta.

[22] Plebani M, Bernardi D, Basso D, Borghesan F, Faggian D (1998). Measurement of specific immunoglobulin E: intermethod comparison and standardization. Clin Chem.

[23] Cox L (2011). Overview of serological-specific IgE antibody testing in children. Curr Allergy Asthma Rep.

[24] Akobeng AK (2007). Understanding diagnostic tests 3: receiver operating characteristic curves. Acta Paediatr.

[25] Graham F, Bégin P, Paradis L, Lacombe-Barrios J, Paradis J, Des Roches A (2016). Comparison of ImmunoCAP and Immulite serum specific IgE assays for the assessment of egg allergy. Allergy Asthma Clin Immunol.

[26] Lee J-HJ-S, Park KH, Kim H-S, Kim KW, Sohn MH, Kim C-H, Lee J-HJ-S, Hong C-S, Park J-W (2012). Specific IgE measurement using AdvanSure^®^ system: comparison of detection performance with ImmunoCAP^®^ system in Korean allergy patients. Clin Chim Acta.

